# Supplementary material for: Effects of Dietary Supplementation with Protected Sodium Butyrate on Gut Microbiota in Growing-Finishing Pigs
Source: Animals (Basel). 2021 Jul 19;11(7):2137. doi: 10.3390/ani11072137 (PMC8300649; doi:10.3390/ani11072137)
Supplement: Supplementary file 1 [file animals-11-02137-s001.zip › animals-1294560-supplementary.pdf]

Table S1. Ingredient and nutrient contents of the three basal diets.

Ingredients (%)	Starting feed	Growing feed	Finishing feed
Maize	20.000	37.278	36.113
Wheat	30.000	28.698	35.000
Soy	20.609	13.032	10.448
Barley	14.303	-	-
Sunflower expeller	-	8.000	8.000
Biscuit cereal meal	10.000	8.000	6.000
Fat mix	1.822	1.704	1.511
Calcium carbonate ($CaCo_3$)	0.794	0.760	0.890
L-Lysine	0.751	0.882	0.692
Dicalcium phosphate	0.586	0.697	0.571
Sodium chloride	0.300	0.300	0.282
Vitamin-mineral premix*	0.200	0.200	0.200
L-Threonine	0.174	0.178	0.188
Sodium bicarbonate	0.130	-	-
Xylanase	0.100	0.100	0.100
Liquid MHA-FA	0.194	0.122	0.047
Phytase	0.015	0.015	0.015
L-Tryptophan	0.017	0.029	0.009
**Nutritional parameters (%)**			
Moisture	12.376	12.325	12.269
Crude protein	17.431	16.050	15.050
Crude fat	4.300	5.010	4.667
Linoleic acid	1.058	1.401	1.332
Crude fiber	3.287	4.120	4.315
Acid detergent fiber	4.314	5.207	5.260
Neutral detergent fiber	11.670	12.577	12.928
Starch	43.445	45.549	47.948
Sugar	4.265	3.712	3.429
Ash	4.596	4.256	4.135
Calcium	0.600	0.600	0.600
Digestible calcium	0.222	0.234	0.232
Total phosphorous	0.465	0.512	0.482
Available phosphorous	0.211	0.226	0.202
Digestible phosphorous	0.280	0.280	0.260
Phytic phosphorous	0.221	0.254	0.252
Potassium	0.705	0.655	-
Sodium	0.225	0.184	0.170
Chlorine	0.278	0.270	0.250
Chlorides	0.467	0.451	0.417
Electrolyte balance	200.000	171.616	160.764
Lysine	1.179	1.092	0.937
Methionine	0.407	0.370	0.294
Methionine-Cystine	0.707	0.651	0.570
Threonine	0.768	0.717	0.627
Tryptophan	0.219	0.204	0.175
Digestible protein	15.045	13.750	13.000
Digestible lysine	1.080	1.000	0.850
Digestible methionine	0.380	0.341	0.273

Table S1. *Cont.*

**Nutritional parameters (%)**	**Starting feed**	**Growing feed**	**Finishing feed**
Methionine + Cysteine	0.637	0.580	0.501
Digestible threonine	0.680	0.630	0.544
Digestible tryptophan	0.194	0.180	0.153
Digestible leucine	1.154	1.083	1.072
Digestible isoleucine	0.614	0.541	0.522
Digestible valine	0.690	0.625	0.604
Digestible phenylalanine	0.725	0.643	0.622
Digestible tyrosine	0.511	0.442	0.430
Phenylalanine + Tyrosine	1.236	1.085	1.053
Digestible histidine	0.379	0.346	0.339
**Net energy (Mcal/kg)**	2.375	2.424	2.421

*Provided per kilogram of complete diet: 3,250,000 IU vitamin A, 750,000 IU vitamin D_3, 7.5 g vitamin E, 1.5 g vitamin B_2, 500 mg vitamin B_6, 8.5 mg vitamin B_{12}, 7.5 g nicotinic acid, 4 g calcium pantothenate, 50 g choline chloride, 50 g Zn, 25 g Mn, 125 g Fe, 5 g Cu, 100 mg Se, 1 g butylated hydroxytoluene, 500 mg I.
